# Evidence Synthesis in Harm Assessment of Medicines Using the Example of Rosiglitazone and Myocardial Infarction

**DOI:** 10.3389/fmed.2017.00228

**Published:** 2018-02-22

**Authors:** Charlotte Rietbergen, Gudrun Stefansdottir, Hubert G. Leufkens, Mirjam J. Knol, Marie L. De Bruin, Irene Klugkist

**Affiliations:** ^1^Department of Methodology and Statistics, Faculty of Social and Behavioural Sciences, Utrecht University, Utrecht, Netherlands; ^2^Astellas Pharma (Europe), Leiden, Netherlands; ^3^Division of Pharmacoepidemiology and Clinical Pharmacology, Utrecht Institute for Pharmaceutical Sciences, Utrecht, Netherlands; ^4^National Institute for Public Health and the Environment, De Bilt, Netherlands; ^5^Copenhagen Centre for Regulatory Science (CORS), University of Copenhagen, Copenhagen, Denmark; ^6^Measurement and Data Analysis, Behavioural, Management and Social Sciences, Research Methodology, University of Twente, Enschede, Netherlands

**Keywords:** evidence synthesis, Bayesian inference, informative priors, benefit vs. risk, safety

## Abstract

The current system of harm assessment of medicines has been criticized for relying on intuitive expert judgment. There is a call for more quantitative approaches and transparency in decision-making. Illustrated with the case of cardiovascular safety concerns for rosiglitazone, we aimed to explore a structured procedure for the collection, quality assessment, and statistical modeling of safety data from observational and randomized studies. We distinguished five stages in the synthesis process. In Stage I, the general research question, population and outcome, and general inclusion and exclusion criteria are defined and a systematic search is performed. Stage II focusses on the identification of sub-questions examined in the included studies and the classification of the studies into the different categories of sub-questions. In Stage III, the quality of the identified studies is assessed. Coding and data extraction are performed in Stage IV. Finally, meta-analyses on the study results per sub-question are performed in Stage V. A Pubmed search identified 30 randomized and 14 observational studies meeting our search criteria. From these studies, we identified 4 higher level sub-questions and 4 lower level sub-questions. We were able to categorize 29 individual treatment comparisons into one or more of the sub-question categories, and selected study duration as an important covariate. We extracted covariate, outcome, and sample size information at the treatment arm level of the studies. We extracted absolute numbers of myocardial infarctions from the randomized study, and adjusted risk estimates with 95% confidence intervals from the observational studies. Overall, few events were observed in the randomized studies that were frequently of relatively short duration. The large observational studies provided more information since these were often of longer duration. A Bayesian random effects meta-analysis on these data showed no significant increase in risk of rosiglitazone for any of the sub-questions. The proposed procedure can be of additional value for drug safety assessment because it provides a stepwise approach that guides the decision-making in increasing process transparency. The procedure allows for the inclusion of results from both randomized an observational studies, which is especially relevant for this type of research.

## 1 Background

The current system of harm assessment of medicines has been criticized as it primarily relies on intuitive expert judgment (1) and there is a call for more quantitative approaches and transparency (2). With respect to the risk-arm of the benefit–risk balance, safety information from different sources accumulates throughout the life cycle of the products (3, 4). At market approval, information on adverse drug reactions (ADRs) of drugs comes from pre-clinical studies and randomized controlled trials (RCTs) whereas post-marketing data mostly include spontaneous ADR reports and epidemiologic studies. Regulators base their pharmacovigilance decisions on both pre-marketing and post-marketing data, which can be conflicting and of deviating relevance and quality, and hence difficult to integrate into a single judgment.

A typical example of a product where information on (cardiovascular) safety accumulated throughout the products life cycle has caused an ongoing debate is rosiglitazone (5–7). Rosiglitazone is an insulin sensitizer used to treat diabetes type II, which was approved by the United States Food and Drug Administration (FDA) in 1999 and by the European Medicines Agency (EMA) in 2000. Subsequently, rosiglitazone was suspended from the European market by the EMA in 2010 due to cardiovascular risk, while it still remains marketed in the United States under severe restrictions (7, 8). The decision to withdraw rosiglitazone from the EU market was based on data that accumulated during the post-marketing phase through use in the general population, which invariably differs from the trial population. The different labels of rosiglitazone in Europe and the US and subsequent market withdrawal in Europe shows how, among others, the evaluation of evidence indifferent regulatory systems can lead to different decisions. Discrepancies such as these occur often and the regulatory systems could benefit from a structured approach to come to a more consistent conclusion.

For integrating information from different sources, post-marketing safety evaluation could benefit from an evidence synthesis strategy for data from both randomized controlled trials (RCTs) and observational studies, especially in combination with judgments of quality and relevance to enable overt combining of data from different sources. Previously, some efforts have been made to combine information from RCTs and observational studies (9, 10). Bayesian statistics can be a useful tool, since data from RCTs and observational studies can either be jointly modeled to estimate an effect, or the observational data can serve as input for the specification of prior distribution for the analysis of the RCT data, or the other way around.

The aim of this paper is to provide a structured procedure for data gathering and quality assessment to combine safety data from RCTs and observational studies. We used the cardiovascular safety of rosiglitazone as an example. With this, we aim to add to the operationalization of the framework provided by Coplan et al. (1) and to provide the regulators with a tool to structure the decision-making when data from many sources are available.

## 2 Methods

**Figure 1:**
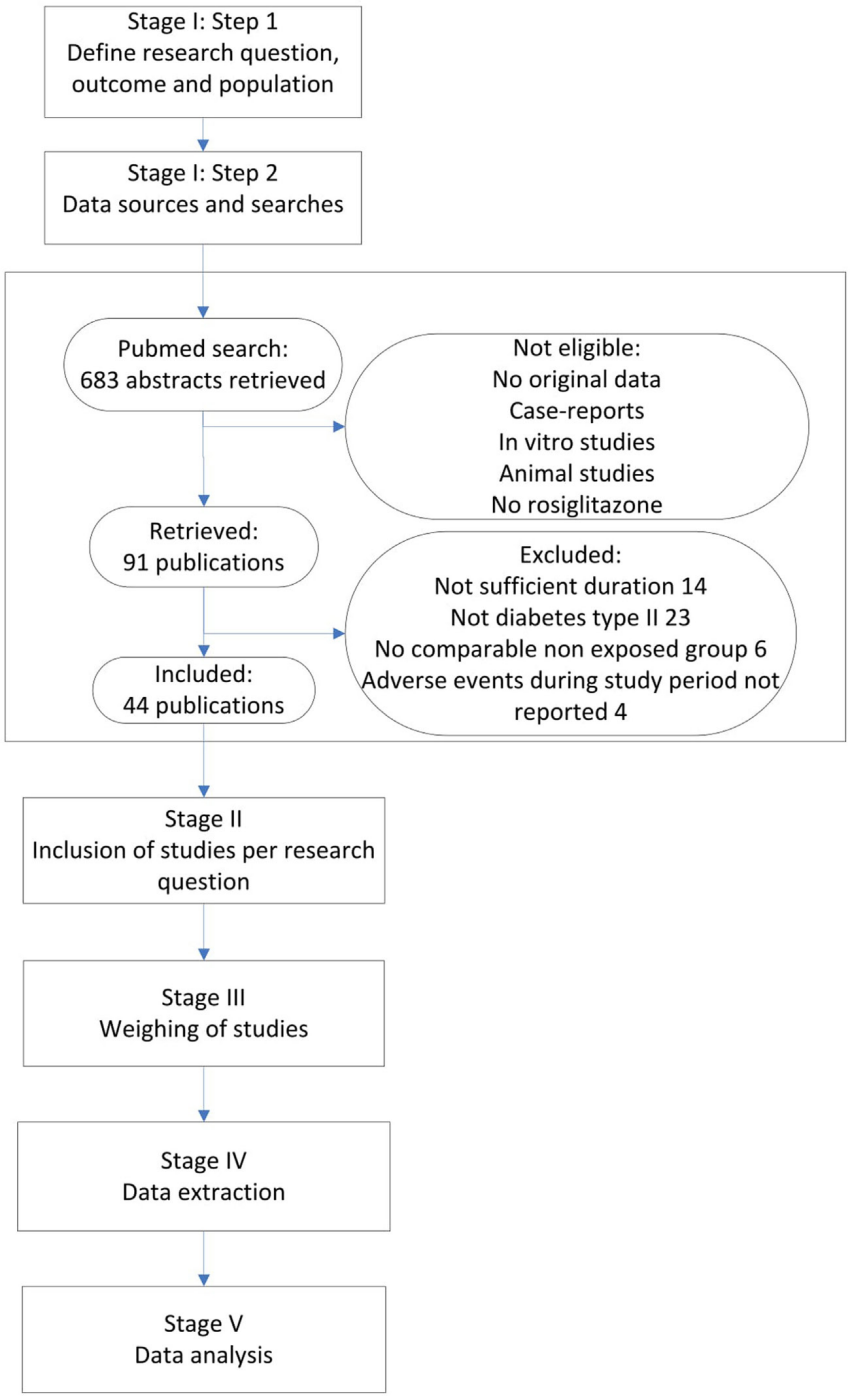
Flow diagram of Rosiglitazone studies.

Figure 1 presents our integrated approach comprising five stages for searching and combining relevant study results from different sources. In the following, we elaborate on each stage of the process and at the same time apply this approach to the rosiglitazone example.

### 2.1 Stage I

#### 2.1.1 Step 1: Defining the Research Question, Population, and Outcome

The assessor has to clearly specify the main research question that one is interested in, and with that, the outcome and population of interest. A preliminary literature search at this point can aid the decision-making with respect to these elements.

Based on information from the literature and the different conclusions about the safety of rosiglitazone, we posed the following overall research question, outcome definition, and population description: Does rosiglitazone increase cardiovascular risk in otherwise healthy adult patients with type II diabetes? A quick scan of several available studies on this topic revealed a great variety in the interpretation of the specified outcome. Some studies reported the total number of myocardial infarctions, strokes, and cardiovascular deaths. Some reported only on one of these major adverse cardiovascular events (MACE). We decided to focus on myocardial infarction (MI) only since the majority of the studies so far reported information on this specific event. With respect to the treatment conditions, all possible comparators were considered and listed, i.e., placebo, no treatment, and other diabetic agents. However, some studies did not report the use of any control group. These studies, in which rosiglitazone was not compared with any other treatment or placebo, were excluded at this stage since these could not contribute to answering the research question.

#### 2.1.2 Step 2: Data Sources, Searches, and General Inclusion Criteria

The inclusion and exclusion criteria have to be listed, and a proper query to search for relevant publications has to be specified.

We performed a Pubmed search, searching for any randomized controlled trials (RCTs) and observational studies on rosiglitazone among adult patients, published before December 31 2010. All studies that mentioned rosiglitazone in the title or abstract were searched. Furthermore, inclusion criteria were a duration of at least 24 weeks and a valid non-exposed group. Only original research articles in English were considered for inclusion. Furthermore, it was required that the number of MI events during the study period was mentioned in the result section, or that a safety section was included that discussed all major adverse events during the study period, whether MI was mentioned or not. If MI was not mentioned in this section, it was considered to have zero events. Since our domain was patients with type II diabetes that were otherwise healthy, only studies that included patients with diabetes type II were considered for inclusion. The search identified 683 abstracts, after excluding non-eligible studies either with no original data, case-reports, *in vitro* studies, animal studies and/or studies without rosiglitazone, 91 publications were retrieved (see Figure 1). From these publications, we excluded 47 studies in which either the study population did not include patients with type II diabetes (23 studies), the studies were not of sufficient duration (14 studies), there was no valid exposure group (6 studies) and/or adverse events during the study period were not listed (4 studies). Finally, 14 observational studies and 30 RCTs were considered for inclusion (see Figure 1 and separate reference list).

### 2.2 Stage II: Inclusion of Studies per Research Question

Although, the information on the safety outcomes of interest is reported in each of the studies selected in Stage I, important differences between the studies might exist with respect to the exact research questions addressed. Some studies are designed to examine the efficacy of the drug under study compared to placebo or other therapies, while others are designed to directly assess the safety of the drug. As a consequence, simple pooling of all available data ignores the underlying safety questions that can actually be answered with the different studies. Therefore, in Stage II, we propose an approach in which a close inspection of the used study designs is made in order to extract the actual research (sub) questions that are addressed. Subsequently, one should extract from each study only those treatment arms that are relevant for one or more of the specified sub-questions. In doing so, the originally intended comparisons should be retained and one should never extract single study arms from any of the studies. Furthermore, study arm selection may not be influenced by study results, i.e., the selection process should take place without consideration of the study results.

**Figure 2:**
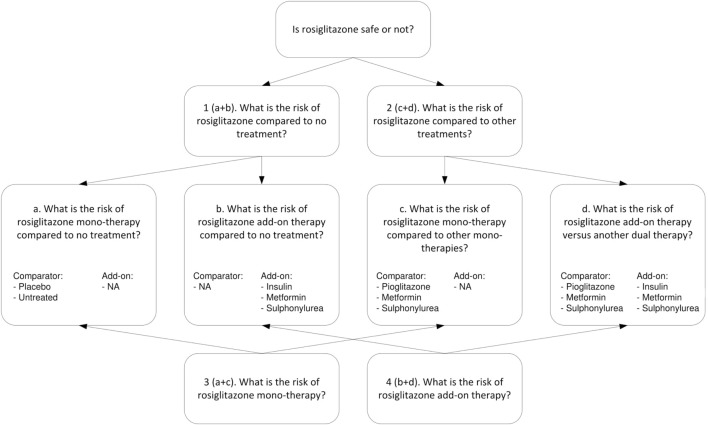
Research questions on the safety of rosiglitazone.

For the example of rosiglitazone, we extracted different types of research questions. Figure 2 shows the four higher level questions (1–4) and four lower level sub-questions (a–d). For each specific research question we assessed, the relevance of the treatment arms and whether the study included a valid non-exposed group. All studies considered for inclusion were reviewed by one of two researchers, and in case of uncertainties reviewed by both. Studies were included only if consensus was reached.

The upper part of Figure 2 addresses research questions 1 and 2. For research question 1, concerning the risk of rosiglitazone compared to no treatment (or placebo), we included (a) studies that had rosiglitazone only arms compared to either placebo or untreated controls (which included observational studies properly adjusted for other glucose control treatments) and (b) studies that evaluated rosiglitazone plus another glucose control agent (rosiglitazone as add-on therapy) vs. the same glucose control agent as monotherapy. For research question 2, which concerned the risk of rosiglitazone compared to other treatments, we included (c) studies that had arms comparing rosiglitazone monotherapy with another monotherapy and (d) studies that compared dual therapy with rosiglitazone and another agent (rosiglitazone as add-on therapy) versus treatment with that same agent plus another glucose control agent (as an add-on).

The lower part of Figure 2 addresses research question 3, about the risk for MI associated with rosiglitazone monotherapy, which is a combination of a and c and research question 4, on the risk of rosiglitazone add-on therapy, which is a combination of b and d.

Table A1 in the Appendix presents a list of all included studies and the selected study arms for each comparison (a, b, c, and d) and the relevant study characteristics. To explain the selection procedure, we take the example of the observational study by McAffee et al. (2007). This study included several treatment arms where rosiglitazone was prescribed both as a monotherapy and add-on therapy. Since rosiglitazone was compared to metformin as well as sulfonylurea monotherapy, the study is listed twice in Table A1 under sub-question c. In addition, rosiglitazone was used as add-on to metformin, sulfonylurea, and insulin and compared to treatment arms where these treatments were used with add-on of sulfonylurea, metformin, and other diabetes agents, respectively. Therefore, these treatment arms were included for sub-question d.

Another example is the RCT by Home et al. (11) which randomized patients on metformin to either rosiglitazone or sulfonylurea and patients on sulfonylurea to either rosiglitazone or metformin. Based on this randomization, we would have included all four study arms in sub-question d. However, in the analysis, the researchers combined both rosiglitazone arms and compared it with both non-rosiglitazone arms. This introduced a problem of whether there was a comparable non-exposed group. What was compared in the end is a group of patients on rosiglitazone and either metformin or sulfonylurea with patients using both metformin and sulfonylurea. Therefore, we concluded that this comparison should be included in sub-question b. Since the results were not adjusted for background medication use (metformin and sulfonylurea), this study was considered less optimal than the observational studies with the same comparisons that do adjust for co-medication.

### 2.3 Stage III: Quality Assessment

**The quality of the evidence synthesis depends on the quality of the individual studies**. An important stage in this procedure is the assessment of quality and relevance of the selected studies. Different scales are available to assess the quality. The Cochrane risk of bias tool for RCTs (12) to assess the weight of randomized studies takes into account method of study treatment allocation and concealment, blinding, completeness of outcome data, and reporting and other sources of bias. The Newcastle–Ottawa quality assessment scale (13) that was designed to assess the risk of bias in case–control studies and cohort studies, consists of three sections that take into account selection, comparability of groups, and exposure in case–control or outcome in cohort studies. The quality scores can be transformed into study weights such that studies with lower quality receive less weight in the meta-analysis and studies of higher quality receive higher weights. In the ongoing debate about the use of quality scores as weights in meta-analysis (14–16), many experts argue that using such weights might induce bias in the estimation of the treatment effect of interest. Since we do attach importance to the process of quality assessment, we propose to use the quality judgment to set a criterion for study (arm) inclusion. For the example of rosiglitazone, we set a cut-off value (lower limit) of 0.7 for studies to be included in our meta-analyses, but different choices in this respect can be made.

We used the abovementioned tools to assess the quality of the included randomized and observational studies in the rosiglitazone example. Since the Cochrane risk of bias tool allows for self-specified potential threats of bias we also included representability of the study population, duration (>24 weeks), and size (>1000 patients). Each study could score 1 point per item on the scale, making up a total of 10 points per study. The final weight is represented as a percentage of the maximum 10 points. The Newcastle–Ottawa scale consists of 3 sections which take into account selection, comparability of groups, and exposure in case–control or outcome in cohort studies. Each study could get a maximum of 9 points. The final score was represented as a percentage of the total 9 points and is presented for each study in Table A2 in the Appendix.

Due to the nature of these scales, studies that are substantially different may receive the same weight. For example, the Newcastle–Ottawa scale allows the user to specify the most important factors that determine the comparability of cases and controls. Each study can earn one point if the included cases and controls match on these factors. A second point can be earned if the study matches cases and controls on additional important factors. We selected age and gender as primary matching factors and diabetic co-medication and previous cardiovascular events as important additional factors. The studies by Dormuth et al. (17) and Dore et al. (18) both received two points for comparability. Unlike Dormuth et al. and many other studies, Dore et al. additionally adjusted for previous diagnosis of obesity and smoking that are important risk factors for cardiovascular disease. The used quality scale, however, does not allow to account for these additional factors.

### 2.4 Stage IV: Data Extraction

In the fourth stage, the focus is on data extraction of general study characteristics, information about the experimental conditions and study outcome. From the randomized studies the number of adverse events per study arm and accompanying size of the arms have to be extracted. In addition, adjusted risk estimates and SEs from all included study arms of the observational studies have to be extracted. The resulting data can be found in Table A2. In this stage, variables that might have influenced the study outcome and, therefore, have to be included as covariates in the final analyses should be considered. For different studies, different variables might be of importance. For some adverse events, the estimated latency time (reported time to event) is much longer than the time needed to measure the efficacy of a drug, hence, the duration of the studies is an important covariate. In other cases, the year of publication might be especially relevant, for example, when there are substantial changes to a drugs label which will affect the population that is being exposed to the drug.

From the randomized trials on rosiglitazone, we extracted information on the absolute number of MI events in all relevant treatment arms and the number of patients in each arm. From the observational study publications, we extracted all adjusted odds ratios (OR) or hazard ratios (HR) and associated 95% confidence intervals for MI (see Table A2). Furthermore, from all included publications we extracted information on publication year, baseline medication (untreated, wash-out period, continued treatment with metformin, sulfonylureas, insulin, or other glucose lowering treatment), comparison treatment (metformin, sulfonylureas, insulin, or other glucose lowering treatment), mean age (in years at baseline), male rate, and duration in weeks. Information was collected at study arm level. Consequently, study information may vary from comparison to comparison.

### 2.5 Stage V: Data Analysis

In the final stage, the data extracted in Stage IV should be arranged per research question, in order to include them in the analyses. Decisions on the statistical models to use have to be made at this stage as well.

For the example of rosiglitazone, we used and compared three models: Model A—a crude analysis of all studies, Model B—a crude analysis of studies with weight ≥0.7, and Model C—an analysis of studies with weight ≥0.7 adjusted for study duration. The rarity of the outcome event of interest in both the treated and untreated (i.e., unexposed to rosiglitazone) patient groups allowed for pooling of odds ratios, risk ratios, and hazard ratios. Therefore, we will use these terms interchangeably.

We performed a Bayesian random effects meta-analyses to pool the observational data per research (sub) question, which consisted of adjusted risk ratios and their 95% confidence intervals. Although observational data are gathered at a later point in time than RCT data, we used the pooled effect estimates of the observational studies, as presented in Table 1, to derive an informative prior distribution per research (sub) question. By adopting a power prior approach (19), it is possible to limit the influence of the observational data on the estimated effect. Which is useful since there is usually much more observational than RCT data available, and the observational data are more likely to produce biased results. By using the power prior, the likelihood of the (pooled) observational data is raised to the power *α*. If this parameter is set to zero, the observational data are fully discounted, while a value equal to one would allow full inclusions of the observational evidence [for a simple introduction on the application of the power prior distribution, we refer to Ref. (20)].

**Table 1:** Results of the Bayesian meta-analysis per sub-questions; prior weight (*α*), mean ES (mean), median ES (med), and lower and upper bounds of the 95% Central Credibility Interval (95% LB and 95% UB, respectively).

		Model A				Model B				Model C			
Question	Prior weight (*α*)	Mean	Median	95% LB	95% UB	Mean	ES median	95% LB	95% UB	Mean	Med	95% LB	95% UB
a	*α* = 0	12.54	0.61	0.01	34.77	14.16	0.59	0	41.33	55.84	0.37	0	123.7
	*α* = 1	1.79	1.71	0.95	3.06	xx	xx	xx	xx	xx	xx	xx	xx
	*α* = 0.0013	6.59	0.67	0.01	32.79	xx	xx	xx	xx	xx	xx	xx	xx
b	*α* = 0	3.48	1.55	0.48	15.94	5.43	1.66	0.44	25.25	7.3	2.54	0.42	35.58
	*α* = 1	1.04	1.04	0.94	1.14	1.04	1.04	0.94	1.14	1.04	1.03	0.94	1.14
	*α* = 0.0002, 0.00006, 0.00003	2.81	1.53	0.48	12.48	4.73	1.64	0.64	23.5	6.52	2.45	0.46	33.74
c	*α* = 0	1.65	1.34	0.47	3.72	1.69	1.35	0.44	3.79	1.38	1.04	0.32	3.48
	*α* = 1	1.05	1.05	0.99	1.12	1.04	1.04	0.95	1.13	1.03	1.03	0.95	1.12
	*α* = 0.0014, 0.0023, 0.0029	1.36	1.3	0.61	2.49	1.37	1.3	0.59	2.56	1.11	1.02	0.43	2.33
d	*α* = 0	3.56	1.73	0.21	16.44	35.72	2.64	0.07	140.9	132.2	2.91	0.02	339.6
	*α* = 1	0.97	0.96	0.83	1.12	0.97	0.96	0.83	1.12	0.97	0.96	0.82	1.12
	*α* = 0.00041, <0.00001, <0.00001	2.97	1.69	0.22	12.47	41.59	2.53	0.06	137.3	287.9	2.98	0.02	287.5
1 (a + b)	*α* = 0	2.25	1.51	0.4	8.4	2.71	1.55	0.35	11.32	3.12	1.88	0.34	12.69
	*α* = 1	1.08	1.07	0.98	1.17	1.04	1.04	0.94	1.14	1.03	1.03	0.94	0.14
	*α* = 0.00051, 0.0003, 0.0002	1.89	1.42	0.39	6.12	2.26	1.5	0.37	8.67	3.05	1.87	0.34	12.52
2 (c + d)	*α* = 0	1.65	1.43	0.67	3.81	1.75	1.44	0.63	4.38	1.61	1.22	0.46	4.25
	*α* = 1	1.05	1.04	0.98	1.11	1.03	1.03	0.96	1.1	1.02	1.02	0.95	1.1
	*α* = 0.0015, 0.0014, 0.0014	1.41	1.35	0.73	2.44	1.47	1.39	0.67	2.84	1.33	1.19	0.53	3.01
3 (a + c)	*α* = 0	1.48	1.32	0.34	3.52	1.49	1.32	0.29	3.78	1.27	1.02	0.21	3.69
	*α* = 1	1.07	1.07	1.01	1.14	1.04	1.04	0.95	1.13	1.03	1.03	0.95	1.12
	*α* = 0.0014, 0,0024, 0,0024	1.35	1.29	0.51	2.6	1.35	1.29	0.48	2.66	1.15	1.04	0.36	2.61
4 (b + d)	*α* = 0	3.01	1.74	0.65	12.33	5.94	1.98	0.62	26.53	7.48	3.26	0.69	36.9
	*α* = 1	1.03	1.02	0.95	1.1	1.03	1.03	0.95	1.11	1.02	1.02	0.95	1.1
	*α* = 0.00016, 0.00003, 0.00003	2.54	1.68	0.64	9.37	4.58	1.95	0.63	22.72	6.9	3.22	0.65	32.67

To monitor the size of the influence of the posterior, we ran the analyses for each research question with three different values for *α*. First, we used *α* = 0 to fully ignore the observational data and *α* = 1 to fully include observational evidence. In addition, we determined the size for *α* based on the variance of the estimated treatment effect in the RCTs. That is, we shrunk the size of the weight parameter such that the variance of the pooled effect found in the observational studies was as large as the variance of the pooled effect in the RCTs. Because the full data were available for the randomized studies, the following model was used for the Bayesian random effect meta-analysis for this part of the data [see also (21)]:

$$\begin{array}{l} r_{i}^{C}\sim Bin(n_{i}^{C},\pi_{i}^{C})\\ r_{i}^{T}\sim Bin(n_{i}^{T},\pi_{i}^{T})\\ \mu_{i}=logit\pi_{i}^{C}\\ logit\pi_{i}^{T}=\mu_{i}+\delta_{i}\\ \delta_{i}\sim N(\delta ,\tau^{2}) \end{array}$$

where $r_{i}^{C}$ and $r_{i}^{T}$ are the estimated risk in study *i* in the control group and treatment group, respectively. Furthermore, $\delta_{i}=logit(\pi_{i}^{T}-\pi_{i}^{C})$ is the log-odds ratio in study *i*, which follows a normal distribution with mean *δ* and between-study variance *τ*^2^. Calculating odds ratios for all RCTs required a continuity correction for those studies with empty cells. To decrease the problem of possible swamping of the real effect, 0.1 was added to all cells instead of the usual 0.5, with one exception: the randomized data for research question d were so sparse that 0.5 was added to the cells to enable estimation at all.

Although in each analysis different combinations of studies and study arms were included, the same model was used. The data for all studies and study arms included per analysis are presented in Table A2. In addition to Model B, in Model C, a study level covariate to adjust for the duration of the study was added to the model. These analyses were only conducted for those research questions for which multiple observational studies as well as multiple intervention studies could be included. All analyses were performed using OpenBUGS 3.2.1. and R (code available upon request). We used non-informative prior distribution for all parameters other than the estimated treatment effect.

## 3 Results

Overall, we found 58 treatment arm comparisons from 30 RCTs and 14 observational studies. From these studies, we included 7 study arm comparisons for research question a (1 observational and 6 RCTs), 16 study arm comparisons for research question b (1 observational and 15 RCTs), 21 study arm comparisons for research question c (13 observational and 8 RCTs), and 14 study arm comparisons for research question d (8 observational studies and 6 RCTs). The majority of the patients included in the trials were men above 50 years of age. Nearly half of included study arm comparisons had duration between 24 and 52 weeks (28 comparisons, 48.3%); consequently, the overall duration of exposure to rosiglitazone was relatively short considering that diabetes is a chronic condition requiring long term treatment. In the first years after marketing of rosiglitazone, only randomized studies were found as expected, from 2007 onward we found publications of observational studies as well. The characteristics of included study arm comparisons per research question can be seen in Table A1 in the Appendix.

The number of MIs in the randomized studies and the adjusted risk estimates (hazard ratios and odds ratios) along with the risk of bias weights can be found in Table A2. Overall, few events were observed in the randomized studies, many did not report any events of MI. RCTs of longer duration such as the one by Home et al. (11) and Kahn et al. (22) reported MI events in both patients exposed to rosiglitazone and the comparison group.

Table 1 presents the results per model for each research question. For each model, we present the results for analysis with prior weights *α* = 0, *α* = 1 and with *α* chosen such that the precision in the observational studies is as large as in the RCTs. By means of this sensitivity analysis, we could evaluate the influence of the prior on the posterior estimates. For research question a, we could not present results for the analysis in which we only included high-quality studies, since only one observational study was available, and this study was of poor quality.

The estimates for the models in research questions a, b, d, 1, and 4 gave very unstable results when no or little prior information was taken into account. Although the estimated mean effect sizes were sometimes large, the associated credibility intervals were so large that we cannot interpret the point estimates. This problem is caused by the fact that the included studies for these research questions reported very few cases of MI. Interestingly, for research question a, all MI events in the rosiglitazone arms of the randomized studies came from the same study. This study is characterized by its long duration (52 weeks) and an exclusive inclusion of patients with a history of cardiovascular disease. Notably, the older studies (published in 2005 or earlier) are shorter than the ones published later. However, the results for Model C as presented in the last column of Table 1 indicates that adjusting for study duration did not noticeably change the results. This result was supported by a simple plot of the effect sizes against study duration, which showed no relationship between the two.

Research question c (rosiglitazone monotherapy versus any other monotherapy) was the only question for which we could include a number of studies in which a substantive amount of MI cases were reported in both the control and treatment arms of the trials. Therefore, only for research question c and for the two questions including c, that is 2 and 3, we found stable results for the meta-analysis for the RCTs. No significant effects were found for research question c and 2 and the majority of models for question 3, meaning that we did not find support in these data for the expectation that patients on rosiglitazone monotherapy are at higher risk for MI than patients on any of the other monotherapies. Nevertheless, the results in Table 1 show how including more prior information pulls the effect size from the estimated effect in the RCTs toward the overall effect size found in the observational studies. At the same time, including more information reduces the size of the 95% credibility intervals, indicating that including prior information provides more confidence in the estimated effect. Take, for example, research question c model C, here the estimated odds ratio found in the meta-analysis of the RCTs alone was found to be equal to 1.38 with 95% credibility interval between 0.32 and 3.48. Adding some information obtained in the observational data pulls the estimated mean in the direction of the OR equal to 1.03 as found in the observational studies, resulting in a posterior mean equal to 1.11. At the same time, the 95% interval was reduced to an interval closer around 1 with a lower bound equal to 0.43 and upper bound equal to 2.33.

Only for research question 3 with Model A, we found a borderline significant effect of 1.07 with 1.01–1.14 95% CI in case the observational data were fully included. This research question asked whether patients receiving rosiglitazone monotherapy were at higher risk for MI compared to patients receiving no treatment (a) or patients receiving any other monotherapy. According to this estimate, we could conclude there is some evidence that patients receiving rosiglitazone only are at somewhat higher risk. However, by using only a small amount of observational information (*α* = 0.0014), or by omitting the low-quality observational studies and RCTs from the analysis, we could not reproduce this significant effect and found the lower bounds of the credibility intervals shifting to <1.

## 4 Discussion

In this paper, we propose a procedure for drug risk assessment which may be used by regulators or researchers that work in the field of pharmacovigilance. The objective of the procedure was to guide and formalize the process of post-marketing safety evaluation and decision-making. We proposed a five-stage approach with which results from carefully selected Phase III and Phase IV studies can be combined. Using rosiglitazone as an example in a meta-analysis, offered a case study to clarify the proposed procedure and to illustrate the difficulties that can be encountered when evaluating safety.

One of the first difficulties we encountered in the first stage had to do with the choice of outcome measure. Initially, we were interested in all major adverse cardiovascular events (MACE) which includes MI, stroke, and cardiovascular death. However, we learned that the included studies reported these adverse events inconsistently. Some studies reported only MIs, others also reported stroke and cardiovascular death. Also, it may not be clear which patients had either MI or stroke and later cardiovascular death or whether the same patient had both MI and stroke, hence, there is a risk of counting these patients twice. Furthermore, the definition of MACE was not homogeneous between the studies, a problem already described by Kip et al. (2008). Therefore, we decided to exclusively focus on MI for the rosiglitazone example. We acknowledge however, the partially spontaneous nature of MI reports in the included studies.

With respect to the quality of the individual studies, we set a cut-off value for studies to be included in our meta-analyses. As said, different choices in this respect can be made, and we advise researchers to perform sensitivity analyses to evaluate the influence of those choices on the result of the evidence synthesis.

An important and distinguishing part of the procedure is discussed in the second stage, where we zoom in on the research questions that underlie the study arms in each randomized and observational study. Selecting the proper study arms and considering the underlying research questions can be quite challenging at times. Many studies use rather complex study designs, and sometimes even propose a different design in the method section than what is actually reported in the result section. An example of this is the study by Home et al. on which we elaborated in the method section and that has been previously criticized for the adjudication of the outcome (23, 24).

Another difficulty that was encountered in Stage II has to do with the fact that study arms were selected for inclusion in the analysis for lower level sub-questions, which were re-used for the analysis at the higher level question as well. Although, we are aware of the fact that with this approach some rosiglitazone patients will be counted twice we consider this the best approach available at this point because of the importance of identifying the right non-exposed group for each sub-question. Another approach may, for example, be to down weigh all arms from the same study so that together the weight of these comparisons equals that of one study.

Except for question 3 under Model A, we did not find a significant association between rosiglitazone and MI for any of our comparisons, which is possibly due to the overall lack of events. For the observational studies, it should be remarked that there might be underreporting of adverse events since the reporting of these events is done on a voluntary basis. From the reported adverse events in the observational studies, we learned that the mean time to event was relatively long, over 1 year. However, most randomized studies were of much shorter duration; for many the follow-up time was between 24 and 28 weeks, and for the majority the follow-up time was between 24 and <52 weeks (16 out of 24 RCTs, 66.7%). Based on this, we hypothesize that the randomized studies may have been too short to investigate the association between rosiglitazone use and MI, even though we did not observe any difference in risk in the model adjusted for duration. This should be kept in mind while designing any future RCTs that test the safety or efficacy of drugs intended for long-term treatment.

When events of interest are rare, which is often the case with adverse drug reactions, classical meta-analysis methods may not perform well (25, 26). Therefore, Bayesian methods have been discussed as an appropriate alternative (27). Previously, others have successfully combined information from RCTs and observational studies with Bayesian methods and various methods to achieve this have been described (27). It was estimated in one meta-analytic comparison that observational studies do not overestimate the effect size of treatment compared to randomized controlled trials (28). Hence, if we have evidence from properly carried out studies, we should not be hesitant to explore innovative methods to combine these data as it will increase the underlying body of evidence and, hence, the power of the analysis. The quality of the method described above depends on the quality of the systematic search for and transparant selection of relevant publications. Therefore, we advise regulators and researchers to implement Stages I–V in existing protocols for meta-analyses that include (a.o.) measures to avoid and detect reporting bias.

The methodological issues discussed above indicate that the currently proposed tool might need to be further validated before it can be implemented and might not be suitable for all regulatory assessments (e.g., for NCEs often data from multiple sources are not available). The results presented should not be used to draw conclusions regarding the authorization status of the products that were used as an example. Furthermore, it would require additional training of regulators before the tool can be successfully implemented.

## 5 Conclusion

The procedure discussed here can be of additional value for drug safety assessment because it provides a stepwise approach that guides the decision-making in order to increase transparency. With this approach, results from randomized and observational studies that include treatment arms which are relevant for the research question are pooled with a Bayesian meta-analysis. Bayesian meta-analysis can be a useful tool to study drug safety because it provides a flexible way of modeling and is considered appropriate when it is challenging to distinguish background adverse events from adverse reactions.

## Author Contributions

This study was designed by CR, GS, MB, and IK. GS performed the data collection and together with CR drafted the manuscript. CR performed the data analyses. MB, IK, MK, and HL supervised the development of work and provided feedback on the manuscript. All authors read and approved the manuscript.

## Conflict of Interest Statement

The authors declare that the research was conducted in the absence of any commercial or financial relationships that could be construed as a potential conflict of interest. The reviewer BE and handling editor declared their shared affiliation.

## Appendix

**Table A1:** Included studies per research question and study characteristics, with study duration in weeks (Duration) and exposure duration in weeks (Exposure).

Year	1st Author	Type of study	Comparator	Add-on	Duration	Mean age	% Male	Exposure
**(a) In patients on rosiglitazone monotherapy compared to no treatment (includes placebo)**								

2001	Lebovitz	Randomized	Placebo		26	60.0	65.7	26
2001	Phillips	Randomized	Placebo		26	57.5	63.4	26
2005	Wang	Randomized	Placebo		26	61.2	82.9	26
2007	Lipscombe	Observational	Adjusted		198	73.9	39.3	197.6
2007	Dargie	Randomized	Placebo		52	64.1	81.2	52
2009	Finn	Randomized	Placebo		35	62.6	76.9	33.6
2010	Bertrand	Randomized	Placebo		52	64.6	91.2	52

**(b) In patients on rosiglitazone add-on therapy compared to the same add-on therapy alone**								

2000	Wolffenbuttel	Randomized	Placebo	Placebo	26	61.2	58.5	26
2000	Fonseca	Randomized	Metformin	Metformin	26	58.2	68.1	26
2001	Raskin	Randomized	Insulin	Insulin	26	56.8	55.6	26
2002	Gomz-Perez	Randomized	Metformin	Metformin	26	53.1	25.7	26
2003	Barnett	Randomized	Sulfonylurea	Sulfonylurea	26	54.2	77.5	26
2003	Zhu	Randomized	Sulfonylurea	Sulfonylurea	24	58.9	44.8	24
2004	Raskin	Randomized	Other	Other	24	57.6	57.6	24
2004	Kerenyi	Randomized	Sulfonylurea	Sulfonylurea	26	59.9	58.5	26
2005	Wong	Randomized	No treatment	Insulin	24	62.3	NA	24
2005	Sarafidis	Randomized	Sulfonylurea	Sulfonylurea	26	62.8	45.0	26
2005	Bailey	Randomized	Metformin	Metformin	24	57.9	57.6	24
2007	Yilmaz	Randomized	Insulin	Insulin	26	59.8	44.1	24
2007	Davidson	Randomized	Sulfonylurea	Sulfonylurea	24	52.5	38.5	24
2008	Chou	Randomized	Sulfonylurea	Sulfonylurea	28	54.1	58.7	28
2008	Koro	Observational	Pioglitazone	Pioglitazone	110	55.5	55.1	109.2
2009	Home	Randomized	Any treatment	Any treatment	260–364	58.4	51.6	286

**(c) In patients on rosiglitazone monotherapy vs. another monotherapy**								

2004	Raskin	Randomized	Other		24	57.6	57.6	24
2004	Baksi	Randomized	Sulfonylurea		26	61.5	60.1	26
2005	Hanefeld	Randomized	Sulfonylurea		52	60.4	65.6	52
2006	Kahn	Randomized	Metformin		208	56.9	57.7	208
2006	Kahn	Randomized	Sulfonylurea		208	56.9	57.7	208
2007	Gerrits	Observational	Pioglitazone		65	58	58	67.6
2007	McAfee	Observational	Metformin		260	52	55	57.2
2007	McAfee	Observational	Sulfonylurea		260	52	55	57.2
2008	Chou	Randomized	Sulfonylurea		28	53.3	58.8	28
2008	Winkelmeyer	Observational	Pioglitazone		31	76.3	26.2	30.7
2009	Dore	Observational	Other		52	66	32.12	54.3
2009	Juurlink	Observational	Pioglitazone		32	73	52.7	47.1
2009	Tzoulaki	Observational	Metformin		364	66.2	50.6	369.2
2009	Rosenstock	Randomized	Other		104	54.3	56.5	104
2009	Hsiao	Observational	Sulfonylurea		48	60.7	54.1	48
2009	Ziyadeh	Observational	Pioglitazone		38	53.3	57.5	40.3
2009	Stockl	Observational	Pioglitazone		234	73	53.9	103.4
2009	Hsiao	Observational	Metformin		48	58.9	45.3	48
2010	Graham	Observational	Pioglitazone		156	74.4	59.8	15
2010	Bilik	Observational	Pioglitazone		76	58.5	45.6	76
2010	Gerstein	Randomized	Other		78	61	67.9	78.1

**(d) In patients on rosiglitazone add-on therapy vs. another glucose lowering agent as an add-on**								

2004	Derosa	Randomized	Pioglitazone	Other	52	53.5	49.4	50.4
2005	Derosa	Randomized	Other	Metformin	52	53.0	50.5	50.4
2005	Weissman	Randomized	Metformin titration	Metformin	24	55.6	NA	24
2007	McAfee	Observational	Sulfonylurea	Metformin	260	52.0	59	62.4
2007	McAfee	Observational	Metformin	Sulfonylurea	260	52.0	59	62.4
2007	McAfee	Observational	Other	Insulin	260	52,0	59	62.4
2007	Yilmaz	Randomized	Metformin	Insulin	24	57.7	43.8	24
2007	Yilmaz	Randomized	Other	Insulin	24	60.1	50	24
2009	Dormuth	Observational	Sulfonylurea	Metformin	520	66.0	73.9	66.0
2009	Dormuth	Observational	Pioglitazone	Metformin	520	68.3	66.4	68.3
2009	Hsiao	Observational	Pioglitazone	Metformin	48	58.9	45.3	48
2009	Hsiao	Observational	Pioglitazone	Sulfonylurea	48	60.7	54.1	48
2009	Hsiao	Observational	Pioglitazone	Other	48	55.5	52.7	48
2009	Raskin	Randomized	pioglitazone	Other	26	55.2	54.3	26

**Table A2:** Reported adjusted effectsizes (ES) and 95% Confidence Interval (95% CI); or number of MI events and groups size (n); and assigned study weights for observational and randomized studies per research question.

Study type	1st Author	ES		95% CI		Weight
**(a) In patients on rosiglitazone monotherapy compared to no treatment (or placebo)**						

Observational	Lipscombe	1.76		[1.27; 2.44]		0.60
		Rosiglitazone		Comparator		
		Events	n	Events	n	Weight
Randomized	Lebovitz	0	335	0	158	0.80
	Phillips	0	735	0	173	0.80
	Finn	0	32	2	33	0.80
	Dargie	5	108	0	110	0.80
	Bertrand	0	98	1	95	0.80
	Wang	0	35	0	35	0.40

**(b) In patients on rosiglitazone add-on therapy compared to the add-on therapy**						

Observational	Koro	1.03		[0.93;1.12]		0.90
		Rosiglitazone		Comparator		
		Events	n	Events	n	Weight
Randomized	Raskin	0	62	0	63	0.60
	Wolfenbuttel	0	382	0	192	0.70
	Fonseca	0	239	0	116	0.90
	Raskin	0	209	0	104	0.90
	Zhu	1	425	0	105	0.80
	Gomz-Perez	0	71	0	34	0.80
	Barnett	1	84	0	87	0.80
	Kerenyi	0	165	0	170	0.80
	Chou	0	442	0	222	0.80
	Wong	0	26	0	26	0.30
	Yilmaz	0	15	0	19	0.40
	Bailey	1	288	0	280	0.90
	Davidson	1	116	0	117	0.80
	Home	64	2,220	56	2,227	0.70
	Sarafidis	0	20	0	20	0.70

**(c) In patients on rosiglitazone monotherapy vs. another monotherapy**						

Observational	Hsiao	1.49		[0.99; 2.24]		0.80
	Hsiao	2.09		[1.36; 3.24]		0.80
	Bilik	0.75		[0.33; 1.67]		0.80
	Dore	1.04		[0.72; 1.51]		0.70
	Ziyadeh	1.35		[1.12; 1.62]		0.70
	Graham	1.06		[0.96; 1.18]		0.60
	Juurlink	0.95		[0.81; 1.11]		0.80
	Tzoulaki	0.79		[0.41; 1.53]		0.80
	Gerrits	0.78		[0.63; 0.96]		0.80
	Winkelmeyer	1.08		[0.93; 1.25]		0.60
	McAfee	1.19		[0.84; 1.68]		0.70
	McAfee	0.79		[0.58; 1.07]		0.70
	Stockl	0.93		[0.72; 1.21]		0.80
		Rosiglitazone		Comparator		
		Events	n	Events	n	Weight
Randomized	Raskin	0	62	0	63	0.60
	Hanefeld	0	384	0	203	0.80
	Baksi	0	225	0	241	0.70
	Kahn	25	1,456	21	1,454	1.00
	Kahn	25	1,456	15	1,441	1.00
	Chou	0	230	0	222	0.80
	Rosenstock	0	202	0	396	0.80
	Gerstein	8	333	7	339	0.70

**(d) In patients on rosiglitazone add-on therapy vs. another glucose lowering agent as an add-on**						

Observational	Hsiao	0.69		[0.3; 1.55]		0.80
	Hsiao	6.34		[1.8; 22.31]		0.80
	Hsiao	1.04		[0.73; 1.47]		0.80
	McAfee	0.41		[0.16; 1.04]		0.70
	McAfee	1.45		[0.76; 2.75]		0.70
	McAfee	0.79		[0.46; 1.36]		0.70
	Dormuth	0.90		[0.69; 1.17]		0.80
	Dormuth	1.00		[0.67; 1.49]		0.80
		Rosiglitazone		Comparator		
		Events	n	Events	n	Weight
Randomized	Derosa	0	42	0	47	0.90
	Derosa	0	48	0	47	0.90
	Weissman	2	358	0	351	0.90
	Yilmaz	0	15	0	17	0.40
	Yilmaz	0	15	0	15	0.40
	Raskin	0	187	0	187	0.50
